# Going Too Far Is the Same as Falling Short^†^: Kinesin-3 Family Members in Hereditary Spastic Paraplegia

**DOI:** 10.3389/fncel.2019.00419

**Published:** 2019-09-26

**Authors:** Dominik R. Gabrych, Victor Z. Lau, Shinsuke Niwa, Michael A. Silverman

**Affiliations:** ^1^Department of Biological Sciences, Simon Fraser University, Burnaby, BC, Canada; ^2^Frontier Research Institute for Interdisciplinary Sciences, Tohoku University, Sendai, Japan; ^3^Centre for Cell Biology, Development, and Disease, Simon Fraser University, Burnaby, BC, Canada

**Keywords:** KIF1, axonal transport, hereditary spastic paraplegia (HSP), neurodegenarative disease, vesicle trafficking, kinesin

## Abstract

Proper intracellular trafficking is essential for neuronal development and function, and when any aspect of this process is dysregulated, the resulting “transportopathy” causes neurological disorders. Hereditary spastic paraplegias (HSPs) are a family of such diseases attributed to over 80 spastic gait genes (SPG), specifically characterized by lower extremity spasticity and weakness. Multiple genes in the trafficking pathway such as those relating to microtubule structure and function and organelle biogenesis are representative disease loci. Microtubule motor proteins, or kinesins, are also causal in HSP, specifically mutations in Kinesin-I/KIF5A (SPG10) and two kinesin-3 family members; KIF1A (SPG30) and KIF1C (SPG58). KIF1A is a motor enriched in neurons, and involved in the anterograde transport of a variety of vesicles that contribute to pre- and post-synaptic assembly, autophagic processes, and neuron survival. KIF1C is ubiquitously expressed and, in addition to anterograde cargo transport, also functions in retrograde transport between the Golgi and the endoplasmic reticulum. Only a handful of KIF1C cargos have been identified; however, many have crucial roles such as neuronal differentiation, outgrowth, plasticity and survival. HSP-related kinesin-3 mutants are characterized mainly as loss-of-function resulting in deficits in motility, regulation, and cargo binding. Gain-of-function mutants are also seen, and are characterized by increased microtubule-on rates and hypermotility. Both sets of mutations ultimately result in misdelivery of critical cargos within the neuron. This likely leads to deleterious cell biological cascades that likely underlie or contribute to HSP clinical pathology and ultimately, symptomology. Due to the paucity of histopathological or cell biological data assessing perturbations in cargo localization, it has been difficult to positively link these mutations to the outcomes seen in HSPs. Ultimately, the goal of this review is to encourage future academic and clinical efforts to focus on “transportopathies” through a cargo-centric lens.

## Introduction

Neurons are highly polarized cells comprised of morphologically, biochemically, and functionally distinct extensions of the cell body: the axon and dendrites. Establishing and maintaining these unique domains depends on the bidirectional transport of cargos within these microtubule-based structures. Active transport utilizes motor proteins, namely the kinesin superfamily (KIFs) for anterograde transport, and cytoplasmic dynein for retrograde transport. When any aspect of this process is dysregulated the resulting “transportopathy” contributes to neurological disorders, such as Huntington’s, Alzheimer’s, and Parkinson’s disease (Millecamps and Julien, 2013). HSPs are also a family of such transport-related diseases with over 80 spastic gait genes (SPG), specifically characterized by lower extremity spasticity. HSPs may present in both pure and complicated forms where the former is primarily limited to progressive lower-extremity spastic weakness and the latter also includes symptoms such as ataxias and cognitive impairments (Blackstone, 2018). Inheritance patterns can be autosomal dominant, autosomal recessive, X-linked, or of mitochondrial (maternal) inheritance (Hedera, 2018). Dominant *de novo* mutations have also been described (Hedera, 2018). Multiple genes in the trafficking pathway related to microtubule function and organelle biogenesis are representative disease loci, such as Spastin (SPG4) and adaptor protein-4 (AP-4 b1; SPG47), respectively (Hazan et al., 1999; Marras et al., 2016). Notably, kinesins are also causal in HSP, specifically mutations in Kinesin-I/KIF5A (SPG10) and two kinesin-3 family members; KIF1A (SPG30) and KIF1C (SPG58; Blackstone, 2018).

HSP-related kinesin mutations have primarily been documented by clinical symptoms and gross pathology combined with medical imaging such as MRI (Lee J.R. et al., 2015; Roda et al., 2017). Furthermore, many thorough studies have previously detailed the inheritance patterns and gene mutations in KIF5 (Liu et al., 2014) and kinesin-3 family members (Supplementary Table 1; Oteyza et al., 2014; Lee J.R. et al., 2015). The structure and function of these kinesins have also been reviewed at length by Hirokawa et al. (2009b) and Siddiqui and Straube (2017) in addition to how HSP mutations impair motor function (Ebbing et al., 2008; Füger et al., 2012; Oteyza et al., 2014; Lee J.R. et al., 2015; Cheon et al., 2017; Jennings et al., 2017; Dutta et al., 2018). The nature of these mutations lead to loss of motor motility or the inability to pause at sites of capture such as at synapses. To our knowledge, no histopathological and little cell biological data assessing perturbations in cargo localization allowing one to infer how mutations in kinesin-3 contribute to HSPs exist. The goal of this review is to link mislocalization of known kinesin-3 cargos to neuronal dysfunction in HSP.

## Overview of Neuronal Transport

Microtubule-based intracellular transport is required by all eukaryotic cells for proper spatiotemporal delivery of proteins and organelles. Intracellular transport is particularly critical for neurons due to their extreme morphological dimensions, polarity, and need for efficient communication between the cell body and distal processes (Bentley and Banker, 2016). Cytosolic and cytoskeletal proteins, such as neurofilaments, tubulin, and tau are moved from the cell body by slow transport, ranging from 0.2 to 2.5 mm per day (Roy, 2014). Slow transport is an essential aspect to neuronal function, and defects in this process contribute to an array of pathologies including Charcot-Marie-Tooth, amyotrophic lateral sclerosis, and Parkinson’s disease (Yuan et al., 2017). This form of transport is mechanistically distinct utilizing primarily kinesin-1 family members and not kinesin-3 (Hirokawa and Tanaka, 2015). By contrast, membranous organelles are moved to the axon terminals by fast transport, which can exceed 400 mm per day (Hirokawa and Tanaka, 2015). Because the axon is largely devoid of biosynthetic machinery, it relies on anterograde axonal transport to supply axon terminals with cargos such as SVP, DCV, and other Golgi-derived proteins and lipids. Retrograde transport from distal portions of the neuron is of equal importance to prevent accumulation of toxic aggregates by clearing recycled or misfolded proteins (Hinckelmann et al., 2013; Millecamps and Julien, 2013), as well as supporting synapse-cell body communication by signaling endosomes ferrying trophic signals (Olenick and Holzbaur, 2019). This bidirectional intracellular transport is driven by kinesin and cytoplasmic dynein motor proteins that use ATP hydrolysis to provide the energy to transport cargos anterogradely toward the synapse or retrogradely toward the cell body, respectively. Notably, axonal and dendritic transport tends to be conflated in the literature. Although mechanistically related, there are key features that distinguish the axon from dendrites that affect transport. For example, dendrites contain MT of mixed polarity, whereas the axon contain MT of plus-end out orientation. Additionally, post-translational modifications on tubulin monomers have region-specific effects on kinesins. Several recent reviews delineate distinguishing features between axonal and dendritic transport (Maeder et al., 2014; Nirschl et al., 2017; Kelliher et al., 2019).

Although seemingly straightforward, the regulation of transport is extremely complex. For example, the Golgi apparatus is the primary site responsible for the maturation of membrane-bound and secreted proteins, and also the segregation into specific organelles based on sorting signals (Bentley and Banker, 2016). Once transport vesicles are formed, they bind to motors typically utilizing a motor-cargo adaptor that can include small GTPases, scaffolding proteins, or the cargo itself. This step of motor-cargo recruitment is complex as there are dozens of post-Golgi cargos and as many transport motors. Although motors are capable of “multi-tasking,” in that one motor is capable of binding several different cargos, specific motors bind only a subset of cargos (Maday et al., 2014). Furthermore, many neuronal cargos eventually display a polarized distribution, for example, presynaptic proteins are delivered to the axon, and postsynaptic receptors, such as glutamate receptors are trafficked to dendrites (Bentley and Banker, 2016). Thus, neurons tightly regulate this delivery either by indirect transport mechanisms where cargo may travel into both the axon and dendrite, yet fuses and is retained in the correct membrane, which is typical of the axon. By contrast, dendritic proteins are dependent upon directed transport where motors either recognize dendritic MT or are excluded from the axon by a filter located in the axonal initial segment (Gumy and Hoogenraad, 2018). Finally, neuronal cargos may be generated at other locations within the cell. For example, autophagosomes are formed in the distal axon and undergo a series of fluctuating transport dynamics from bidirectional in the distal axon toward a bias of retrograde, dynein based-transport toward the cell body (Stavoe and Holzbaur, 2019). Mitochondria, which are distributed throughout the neuron undergoing fusion, fission, and remodeling, also travel bidirectionally in both axons and dendrites (Saxton and Hollenbeck, 2012). Taken together, HSP-related kinesin mutations, and those found in other disease states, will reduce the efficacy of active transport and perturb cargo delivery, ultimately leading to neuronal dysfunction.

## Kif1A Structure and Function

**FIGURE 1:**
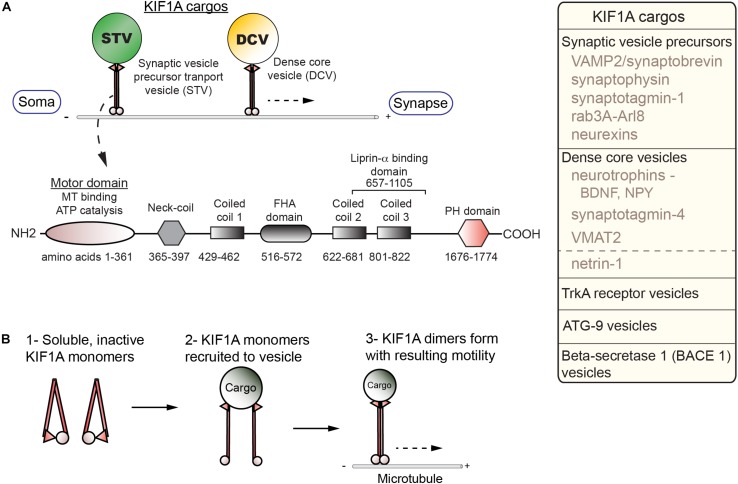
Cargos, structure, and regulation of KIF1A. **(A)** KIF1A is a homodimer that traffics a number of cargos (box; right) from the soma to distal portions of the axon including synapses. Below, a schematic indicates the domains identified in KIF1A (adapted from Soppina et al., 2014a; UniProt #Q12756). **(B)** Diagram of activation steps of KIF1A.

KIF1A carries a number of critical cargos, including SVP, DCVs, and BACE-containing vesicles all of which will be explored in greater depth in the following section (Figure 1A). KIF1A is generally similar in overall structure to other kinesin family members as they contain motor, coiled-coil, and cargo-binding domains. KIF1A, a neuron-enriched motor, belongs to the Walker-type ATPase family: ATPase’s defined by a conserved phosphate-binding loop (P-loop) containing Walker A and Walker B motifs (Hirokawa et al., 2009a). The entire kinesin-3 family motor domain sequence is highly conserved with KIF1A being no exception. The motor domain (MD) is composed of both a catalytic core in which the N-terminal half acts as the ATP catalytic center with the P-loop forming the nucleotide-binding pocket, while the C-terminal half acts as the MT-binding surface (Hirokawa et al., 2009a). The MD is connected via a flexible NL to a neck coil domain (NC), which is followed by the coiled-coil 1 (CC1), forkhead-associated (FHA), coiled-coil 2 (CC2) and coiled-coil 3 (CC3) domains, respectively. There is also a pleckstrin homology (PH) region which acts as a C-terminal lipid-binding domain. Furthermore, KIF1A contains an insert of positively charged lysine residues in loop 12, an area within the MD known as the K-loop, which is also conserved across the kinesin-3 family (Hirokawa et al., 2009a). This region allows for enhanced binding to the C-terminal region of tubulin known as the E-hook (glutamine rich). As a result, KIF1A undergoes super-processive movement making it especially efficient for long-range axonal transport (Soppina et al., 2014a).

Regulation of KIF1A is complex and not fully understood. Competing models have posited that KIF1A is a motile monomer (Okada et al., 1995), an autoinhibited dimer (Hammond et al., 2009; S. Niwa, Personal Communication), or monomers that dimerize on the cargo membrane (Tomishige et al., 2002; Soppina et al., 2014a). The current model demonstrates activation of KIF1A is dependent on a monomer to dimer transition at the cargo surface (Soppina and Verhey, 2014b; Figure 1B). This process involves relief of autoinhibition, a regulatory mechanism seen in other KIFs. The formation of the dimer at the cargo surface depends on an interaction between the NC and CC1 domains. To form a dimer, an intramolecular interaction between the NC and CC1 domains must be relieved (Soppina and Verhey, 2014b). In the case of SVP, relief of autoinhibition involves interactions with the GTPase ARL-8 and CC domains of UNC-104/KIF1A (Niwa et al., 2016). The dimer forms via an intermolecular reaction between the NC domains of the monomers and allows for motility. The catalytic core is regulated by switch II, a region within the C-terminus of the core (Hirokawa et al., 2009a). This works in concert with the NL, with the complex acting as an actuator to produce mechanical work. Switch I, a segment between the P-loop and switch II, links, and therefore regulates, the switch II-NL complex via the nucleotide state of the binding pocket (Hirokawa et al., 2009a).

Cargo binding is an essential aspect to motor-driven transport and has been reviewed extensively (Karcher et al., 2002; Vale, 2003; Ross et al., 2008; Bentley and Banker, 2016). Knowledge on KIF1A cargo binding and how it relates to motor function is still not well characterized, but certain mechanisms have been elucidated. One of the first mechanisms identified for KIF1A cargo interactions entails the C-terminal PH domain binding to phosphatidylinositol-4,5-bisphosphate [PI(4,5)P_2_] on synaptic vesicles in *C. elegans* neurons (Klopfenstein and Vale, 2004). However, protein-protein interactions lend specificity to cargo recognition and regulation. For example, the GTPase Rab3a regulates the binding of SVP to KIF1A in a DENN/MADD dependent mechanism (Niwa et al., 2008). DENN/MADD acts as a Rab3-effector, allowing for transport of SVPs down the axon. A shift in the nucleotide state of Rab3 releases SVPs at the synapse. For DCVs, another KIF1A cargo, a few mechanisms have been described that may account for their trafficking. One such mechanism involves carboxypeptidase E (CPE), a transmembrane protein needed for neuropeptide processing, where it also acts as a cargo adaptor (Park et al., 2008; Ji et al., 2017). The cytoplasmic tail of CPE binds the dynein activator dynactin. Dynactin also recruits KIF1A, thereby creating a mechanism to potentially regulate the bidirectional transport of DCVs. Another mechanism implicated liprin-α as a possible KIF1A adaptor (Shin et al., 2003), however, recent findings indicate that liprin-α, in addition to TANC2, act to capture KIF1A at specific sites (Stucchi et al., 2018). Instead, the PH domain in concert with calmodulin (CaM) dictates binding and movement of DCVs in response to increases in Ca^2+^ concentrations through direct binding to the KIF1A stalk domain (Stucchi et al., 2018). Taken together, mutations that affect anyone of the various cargo-binding and regulatory domains involved in these processes may lead to dysregulation of KIF1A-mediated cargo delivery.

## Kif1A Mutations in Spg-30 and Related Disorders

**FIGURE 2:**
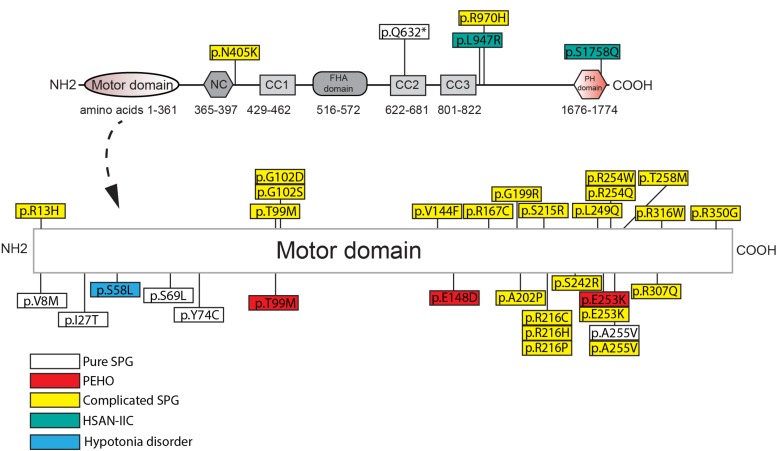
Amino acid changes in KIF1A in HSP and related disorders in humans. See Supplementary Table 1 for details of each mutation. NC, neck-coil; CC, coil-coil; FHA, Forkhead association; PH, Pleckstrin homology.

SPG-30 causing KIF1A mutations are found in the motor, regulatory, and cargo binding regions (Figure 2 and Supplementary Table 1). As such, specific mutations may differentially affect KIF1A function depending on the domain. For example, E253K affects ATPase activity within the motor domain, while the Q632^∗^ deletion mutant removes the CC2, CC3, and PH domains critical for regulation and cargo binding (Esmaeeli-Nieh et al., 2015; Van De Warrenburg et al., 2016). The consequent changes in KIF1A function are ultimately mislocalization of cellular cargos via distinct mechanisms, including failure to properly regulate KIF1A motility and to bind to cargo. The extent and severity of SPG-30 symptomology is dictated by the patient’s genotype and the resulting changes in KIF1A amino acid residues.

Loss-of-function mutations in the motor domain of KIF1A constitute the vast majority of SPG-30 cases. They affect a variety of structural domains such as those critical for ATP hydrolysis, generation of mechanical force (Switch I and II), and microtubule binding (loop L8). Some examples include mutations that reside in Switch I (R216C) and Switch II (E253K), as well as mutants that destabilize loop L8 (R316W) (Esmaeeli-Nieh et al., 2015; Lee J.R. et al., 2015). The vast majority of KIF1A mutants have not been analyzed for function, but a subset results in impaired motility and/or an altered distribution compared to wild type, where the motor accumulates proximally. For example, the Switch II mutant E253K, and the ATP-binding cassette mutant T99M, have drastically reduced motility and are unable to reach the distal portions of the axon (Esmaeeli-Nieh et al., 2015; Cheon et al., 2017). This suggests that the inability to traffic cargos into the axon may underlie downstream pathogenesis in SPG-30. Surprisingly, gain-of-function mutations in KIF1A are also seen in SPG-30. Using single molecule assays, Chiba et al. (2019) recently demonstrated, that three KIF1A mutants causal in SPG-30 (V8M, R350G, A255V) had higher landing rates on MT’s, along with a greater velocity than WT KIF1A in two of the three mutations (A255V had similar velocity to WT). Notably in two of the mutants (V8M, A255V), SVPs accumulated at the axonal tips of *C. elegans* ALM neurons, suggesting that excessive cargo accumulation may be pathogenic.

Moreover, an interplay between a variety of factors influence the pathogenicity of the mutation including the functional domain, the amino acid properties, and heritability patterns. Conserved amino acid changes, such as the A255V mutant, tend to result in less severe effects on motor motility, which is reflected in reduced severity of symptomology (Esmaeeli-Nieh et al., 2015; Cheon et al., 2017). Mutants in which the residue properties are drastically changed, such as that in the E253K mutant, show much more severe motor motility changes, leading to complicated SPG-30 (Esmaeeli-Nieh et al., 2015; Cheon et al., 2017). Some SPG-30 loci have multiple different amino acid changes, for example, R216 mutations can be either a histidine, proline, or cysteine leading to differing clinical symptoms (Lee J.R. et al., 2015; Esmaeeli-Nieh et al., 2015; Travaglini et al., 2018). It is important to note that without histopathological samples, the link between changes in motor properties and symptomology is tenuous. Furthermore, prediction of the presentation of the disease based on the mutation is difficult as certain conserved mutations can differ in the severity of symptom presentation. This is exemplified by the A255V mutant, as it causes both the pure and complicated forms of SPG-30 where a patient’s genetic background may affect how the mutations manifest physiologically (Erlich et al., 2011; Klebe et al., 2012). As such, one cannot say with certainty that the amino acid properties are the sole determinants of how the disorder will present. Larger patient populations would be needed to confirm such a hypothesis. Lastly, it is important to note that mutations implicated in complicated SPG-30, such as T99M and E253K, are causal in PEHO syndrome suggesting a similar etiology between the disorders (Esmaeeli-Nieh et al., 2015; Lee J.R. et al., 2015; Samanta and Gokden, 2019). Furthermore, a mutation exclusive to PEHO syndrome, E148D, presents with symptomology akin to complicated SPG-30, further suggesting that the etiology of these KIF1A-related disorders may be similar (Ohba et al., 2015).

Mutations that implicate regulatory and cargo binding regions of KIF1A are both causal in pure and complicated forms of SPG-30 and HSAN-IIC. Presently, no studies exist that assay the motility, regulatory, or cargo binding capabilities of these KIF1A mutants. However, mutations in the KIF1A PH domain lead to reduced cargo binding in *C. elegans*’ neurons (Klopfenstein and Vale, 2004). Thus, disease loci that likely truncate the cargo binding domains of KIF1A, such as the pure SPG-30 Q632^∗^ mutant, might result in in a complete absence of cargo binding and delivery (Van De Warrenburg et al., 2016). Despite such changes to KIF1A in SPG-30, patients can live for decades with this disorder implying there is some redundancy in KIF1A function. One example may be that DCVs can be transported in tandem or by alternate motors such as KIF1C and KIF5 (Gauthier et al., 2004; Lim et al., 2017; Stucchi et al., 2018). The fidelity of cargo delivery may be altered when the complement of neuronal kinesins are shifted.

## Kif1A Cargos

Although it is proposed that KIF1A mutations lead to cargo mislocalization and ultimately disease, no histological data in animal models or humans currently explains this. We will explore several KIF1A cargos in an attempt to bridge motor mutations, transport aberrations, and clinical symptomology (Figure 4). Where possible, we will also attempt to focus on the impact of cargo mislocalization in specific neuronal subtypes, for example, differences between CNS and PNS neurons.

### Synaptic Vesicle Precursors

The first cargo identified for KIF1A was the SVP, a Golgi-derived organelle containing essential proteins required to build synapses and regulate synaptic vesicle release (Okada et al., 1995). KIF1A/UNC-104 misdelivery of SVPs alters the size and density of synapses in model organisms such as *C. elegans* and mice (Yonekawa et al., 1998; Niwa et al., 2016). SVPs are required in every neuron in the body, thus, KIF1A mutants may have broad effects on HSP patients. KIF1A traffics SVPs involved in various aspects of vesicle fusion and release, in particular, synaptotagmin-1 (syt-1), VAMP2, and synaptophysin (Okada et al., 1995; Yonekawa et al., 1998; Sgro et al., 2013). These proteins are expressed in all cells undergoing synaptic vesicle exocytosis, therefore, any form of disruption may cause widespread synaptic dysfunction. Syt-1 is a membrane-bound Ca^2+^-binding protein that is critical for inhibiting spontaneous fusion of synaptic vesicles and for fast synchronous release of synaptic vesicle contents (Maximov and Südhof, 2005; Bacaj et al., 2013; Lai et al., 2014; Bornschein and Schmidt, 2019). Syt-1 knockout hippocampal neurons lost this ability but retained slow, asynchronous release, whilst release of the neurotransmitter glutamate was maintained at normal levels (Maximov and Südhof, 2005; Bacaj et al., 2013). Interestingly, asynchronous release of the neurotransmitter glutamate results in an increase in postsynaptic activity (Iremonger and Bains, 2007; Iremonger and Bains, 2016). As such, increases in NMDAR opening may follow and cause spikes in local Ca^2+^ concentrations in downstream neurons resulting in neurodegenerative cascades in susceptible populations of neurons (Dong et al., 2009).

VAMP2 is a SNARE protein involved in the formation of the SNARE complex with syntaxin-1 and SNAP-25 (Südhof, 2013). In VAMP2 knockout mouse models, both spontaneous vesicular fusion and Ca^2+^-induced fusion was severely depleted but not ablated (Schoch et al., 2001). Defects in HSP-related KIF1A motility or cargo binding may mirror this knockdown phenotype, resulting in a reduced synaptic vesicle release which may have neurodevelopmental consequences. Interestingly, when the SNARE complex partners SNAP-25 and syntaxin-1 are knocked out, neurons go through a neurodegenerative process, while VAMP2-deficient neurons do not (Schoch et al., 2001; Santos et al., 2017). This suggests that VAMP2 participation in the SNARE complex is not critical for cell viability.

Another KIF1A cargo, synaptophysin, is thought to be an adaptor for VAMP2 endocytotic recycling as well as aid in synaptic vesicle maturation, as the VAMP2/synaptophysin complex cannot fuse (Becher et al., 1999; Schoch et al., 2001). Synaptophysin mutants are implicated in a variety of neurodevelopmental disorders such as X-linked intellectual disability, epilepsy, and hypotonia, similar to what is seen in complicated SPG-30 (Gordon and Cousin, 2013; Harper et al., 2017). As synaptophysin knockouts very closely approximate synaptophysin mutant phenotypes (Harper et al., 2017), namely failure to retrieve VAMP2, it is possible that impaired synaptophysin trafficking by mutant KIF1A may copy such phenotypes.

Synaptic Vesicle protein 2A (SV2A), although previously not thought to be transported by KIF1A, was identified as a cargo by Sgro et al. (2013), Okada et al. (1995). It is involved in regulation of synaptic vesicle release and SV2A deficiencies lead to a disrupted GABA exocytosis contributing to temporal lobe epilepsy (Crowder et al., 1999; Janz et al., 1999; Menten-Dedoyart et al., 2016). As epilepsy is also seen in complicated SPG-30, it is possible that KIF1A mutations contribute to epileptic seizures by mislocalizing and impeding SV2A function.

Lastly, Rab3a, the GTPase that regulates SVP transport by KIF1A in a DENN/MADD-dependant fashion, also plays a role in fusion probability regulation of synaptic vesicles (Geppert et al., 1994, 1997). Furthermore, Rab3a seems to recruit synaptic vesicles to the active zone. Rab3a knockout mice have demonstrated that although the vesicle numbers in the synapse stay the same, recruitment to the active zone is ablated (Leenders et al., 2001). As such, secretion of synaptic vesicular cargos is reduced, a phenotype that should be mimicked with dysfunctional KIF1A. Neurodevelopmental defects as a result of dysregulation in synaptic transmission may be a consequence.

### Neurexins Are SVP Cargos

The neurexins are single-pass transmembrane proteins acting as cell-adhesion molecules that are derived from three genes (*Nrxn 1-3)*. They are split into long α and short β isoforms via two distinct promotors found in each of the three neurexin genes. Alternative splicing may result in thousands of different isoforms. The various neurexins are found throughout the CNS but the expression of the neurexins changes throughout development, as well as the function of particular neurexins can differ in different neuronal populations (Chen et al., 2017; Harkin et al., 2017). They are mainly concentrated at presynaptic sites and broadly function in neurite outgrowth, synaptogenesis, and synaptic maturation (Dean and Dresbach, 2006; Gjørlund et al., 2012; Krueger et al., 2012; Gokce and Sudhof, 2013). This is accomplished through interactions with a plethora of intracellular proteins via their C-terminal PDZ-recognition motif, as well as with extracellular proteins such as the neuroligins via their N-terminus. The various pathways and downstream effects that are elicited through these interactions have been extensively reviewed previously (Südhof, 2017). Furthermore, mutations in neurexins are linked to several neurodevelopmental and psychiatric disorders such as autism, epilepsy, and mental retardation (Kasem et al., 2018). Mouse models of neurexin conditional knockouts show little to no change in number of synapses, but do show presynaptic transmission defects (Missler et al., 2003). However, triple knockout mouse models generated by Chen et al. showed that certain neuronal populations do show a loss of synapses in response to loss of neurexins (Chen et al., 2017).

Trafficking of both α- and β-neurexins anterogradely to distal axonal sites is accomplished by KIF1A-mediated SVP transport (Neupert et al., 2015). In addition, both of these neurexins colocalize with synaptophysin, another known SVP cargo. Knockdown of KIF1A results in reduced delivery of the neurexins to the plasma membrane (Neupert et al., 2015). To our knowledge, there are no follow up studies to demonstrate how reduced neurexin trafficking may affect the development and function of the synapse. However, it is possible that the inability for HSP-related KIF1A mutants to traffic neurexins to the distal membranes may result in similar presynaptic transmission defects as seen in conditional knockouts. Synaptic dysfunction is a critical pathogenic event that often precedes neuronal degeneration and is seen in a variety of other neurodegenerative disorders including Alzheimer’s, Parkinson’s, Huntington’s disease, and ALS (Coleman et al., 2004; Wishart et al., 2006; Morfini et al., 2009; Bae and Kim, 2017). Such synaptic defects may underlie the “dying-back” phenotype that is seen in certain populations of neurons in HSP. Lastly, Chen et al. demonstrated that different hippocampal populations show differential pathological synaptic phenotypes in response to neurexin knockouts which suggests different vulnerabilities (Chen et al., 2017). As such, it is possible that the neurons mainly affected in HSP, namely the upper CST neurons, may be more susceptible to neurexin misdelivery.

### Dense Core Vesicles

The DCV was the second cargo identified for UNC-104/KIF1A in *C. elegans* (Zahn et al., 2004). This finding was subsequently confirmed in *Drosophila*, and rodent models (Barkus et al., 2008; Lo et al., 2011; Kondo et al., 2012), however, KIF1C and KIF5 may also contribute to DCV transport (Gauthier et al., 2004; Lim et al., 2017; Stucchi et al., 2018). Dense-core vesicles (DCVs) are Golgi-derived vesicles typified by the presence of granin family members. DCVs contain a host of proteins necessary for proper neuronal development, function, and viability. Examples of cargos include neurotrophic peptides such as BDNF and NPY, both essential for a multitude of neuronal functions (Dieni et al., 2012; Russo, 2017). An additional cargo of note is POMC, which can be cleaved into a host of physiological important peptides including a-MSH, ACTH, and endorphins (Cawley et al., 2016). Lastly, DCVs contain a host of other cargos including, proteases, protease inhibitors, and membrane proteins such as tPA, neuroserpin, and VMAT2, respectively (Nirenberg et al., 1995; Lochner et al., 1998; Ishigami et al., 2007). Despite the potential heterogeneity of DCVs across different neuron types, they share KIF1A transport mechanisms. As such, dysfunctional KIF1A-mediated DCV transport may lead to pathogenesis in neurons. In this section, we focus on only a few select cargos and their potential involvement in SPG-30 pathogenesis.

### Brain-Derived Neurotrophic Factor

Brain-derived neurotrophic factor is a neurotrophin critical for survival, as knockout mice die shortly after birth (Ernfors et al., 1994). BDNF binds the TrkB and p75_NTR_ receptors activating downstream signaling cascades such as PLCγ1, Ras-MAP kinase, and PI3 kinase to regulate neurotransmission, cytoskeletal and membrane dynamics, and gene transcription, ultimately affecting synaptic development, neuronal plasticity, and survival (Kirschenbaum and Goldman, 1995; Lipsky and Marini, 2007; Nikoletopoulou et al., 2017; Kowiañski et al., 2018). In addition to trans-synaptic signaling, BDNF functions in an autocrine manner at dendritic spines to regulate NMDA receptor – CaMKII signaling, supporting functional and structural aspects of plasticity (Harward et al., 2016). In axons, this autocrine action stimulates a positive feedback loop to enhance BDNF secretion and TrkB trafficking to the membrane to promote axon differentiation and growth (Cheng et al., 2011). BDNF is expressed throughout the nervous system including in the cortex, cerebellum, hippocampus, and pyramidal tracts (Hofer et al., 1990; von dem Bussche and Tuszynski, 2010). Upper motor neurons signal to lower motor neurons to regulate voluntary movement and muscle tone. As HSP is a distal axonopathy, reduced BDNF availability in these neurons might affect their development and survival (Giehl and Tetzlaff, 1996; Lu et al., 2001). Complicated SPG-30 may include cerebellar dysfunction typically characterized by ataxias as cerebellar neurons would be affected similarly to other cell types by the lack of BDNF (Carter et al., 2002). In fact, Mellesmoen et al. (2019) recently demonstrated that endogenously applied BDNF can delay cerebellar dysfunction onset in mouse models of ataxia. Cognitive deficits arising in complicated HSPs may be explained by similar mechanisms. In the hippocampus, BDNF regulates LTP but interestingly not survival (Ip et al., 1993; Leal et al., 2017), similar to neurons of the prefrontal cortex that are required for executive function (Galloway et al., 2008).

### Neuropeptide Y

NPY, another essential neuropeptide is trafficked in DCVs in a KIF1A dependent manner (Lo et al., 2011). NPY governs neurogenesis and neuroprotection, and is expressed in areas associated with HSP such as the cerebral cortex, hippocampus, and cerebellum (Berg et al., 2013; Gøtzsche and Woldbye, 2016). Depleted NPY levels in the cerebellum are associated with progressive motor impairment in mouse models, while increasing levels seems to ameliorate these impairments (Duarte-Neves et al., 2015). This suggests that in complicated SPG-30 with cerebellar involvement, NPY may dictate pathogenesis. In the hippocampus, NPY plays a critical role in neurogenesis through a Y1 receptor-mediated pathway (Howell et al., 2003). As learning and memory is thought to be dependent on the genesis and incorporation of neurons into certain hippocampal circuits (Ramirez-Amaya et al., 2006), failure to deliver and release NPY to target sites may affect these processes. Learning and memory is affected in certain complicated SPG-30 cases, which underscores the possibility of NPY-mediated deficits in this disorder.

### Nerve Growth Factor (NGF) and Neurotrophin-3 (NT-3)

Although there are likely to be numerous other DCV cargos where KIF1A involvement has not been confirmed, it is worth speculating on their potential central role in HSP pathology. For example, secreted peptides such as NGF and NT-3 have differentiation, proliferation, and survival roles in the developing and mature nervous system (Belliveau et al., 1997; Zhu et al., 2012; Martorana et al., 2018). NGF and its receptors (e.g., TrkA, TrkB, p75_NTR_) are found in a wide range of neurons including those within cortical regions of the brain affected in HSP such as the hippocampus, somatosensory and motor cortex, as well as those implicated in complicated forms such as the cerebellum (Pitts and Miller, 1995; Conner et al., 2009). NGF regulates a variety of neuronal processes such as axon outgrowth, and receptivity to myelination (Chan et al., 2004; Turney et al., 2016). It also provides neuroprotection to excitotoxicity, a pathology implicated in a number of neurodegenerative disorders (Kume et al., 2000). Different localizations of NGF within neurons can affect whether neurons will undergo apoptosis or axonal pruning (Geden and Deshmukh, 2016; Geden et al., 2019). When NGF is compartmentalized to the soma and proximal axons (thereby depriving distal axons) axonal pruning occurs, with the opposite resulting in apoptosis-induced axonal and somal degeneration. Certain KIF1A mutations, such as the Switch II and ATP-binding cassette mutants, E253K and T99M, cause a somal/proximal axon localization of cargos and motors which, taken together, may indicate that mislocalization of NGF underlies the Wallerian-like, or “dying-back” phenomena, seen in HSP.

In addition to differentiation, proliferation, and survival roles similar to NGF, NT-3 plays critical roles in synaptic development, organization, and transmission through interactions with the Trk family of receptors (Paul et al., 2001; Han et al., 2016). NT-3 is the most abundantly expressed neurotrophin in the developing CNS, particularly in regions such as the hippocampus, neocortex, cerebellum, and spinal cord, with levels drastically dropping in the adult CNS (Maisonpierre et al., 1990). Interestingly, NT-3 mRNA is primarily found within motor neurons (Ernfors and Persson, 1991), and enhances CST axon collateralization in the developing nervous system (Schnell et al., 1994). Furthermore, the TrkC receptor, which has the highest affinity for NT-3, is highly expressed in regions of the cortex where CST neurons originate (Ringstedt et al., 1993), suggesting that NT-3 is important for inducing CST formation. Neuromuscular synapse maturation and potentiation also depends on NT-3 as Xenopus nerve-muscle co-cultures show increased levels of synaptophysin and synapsin 1 in spinal neurons, and increased frequencies of spontaneous synaptic currents, respectively (Lohof et al., 1993; Wang et al., 1995). Lastly, NT-3 is critical in the survival of TrkC-positive muscle spindle afferents (Oakley et al., 1997). Mouse models homozygous for defective TrkC lacked 1a muscle afferent projections to spinal motor neurons, resulting in defective movement and posture (Klein et al., 1994). Relating this to HSP, reduced transport of NT-3 may therefore affect muscle-related proprioceptive capabilities via muscle spindle afferent dysfunction resulting in the spasticity, possible ataxias related to developmental defects in neurons of the cerebellum, memory and cognitive defects related to hippocampal and neocortex developmental deficits, and weakness due to CST dysfunction.

### Synaptotagmin-IV

The DCV associated synaptotagmin-IV (Syt-IV) belongs to a family of membrane-trafficking proteins, many of which act as Ca_2_^+^-sensors that allow for membrane fusion and binding of SNARE proteins (Südhof, 2002; Dean et al., 2009). Syt-IV cannot bind Ca_2_^+^ directly, however, it reversibly binds SNARE proteins in a Ca_2_^+^-dependent fashion (Wang et al., 2003). Ca_2_^+^ influxes cause release of Syt-IV from the SNARE proteins allowing for a fusion event to occur both pre- and post-synaptically. Syt-IV is distributed throughout the brain with particular enrichment within the hippocampus and cerebellum (Ferguson et al., 2000); two areas affected in KIF1A-dependent complicated HSPs. Notably, colocalization studies between BDNF-containing vesicles and Syt-IV indicate that Syt-IV associates with DCVs (Dean et al., 2009). In Syt-IV knockout mouse models, mice show an increase in spontaneous quantal release of BDNF in pre-synaptic regions, and an increase in LTP, processes that underlie learning and memory (Dean et al., 2009). Other studies confirm effects on both hippocampal-mediated memory and learning, as well as motor coordination defects consistent with cerebellar involvement (Ferguson et al., 2000, 2004). Through both co-immunoprecipitation and trafficking studies, Syt-IV was confirmed to have a direct interaction with KIF1A (Arthur et al., 2010; Bharat et al., 2017). KIF1A knockdowns in hippocampal neurons result in a significant reduction in synaptic vesicles, as well as causing an accumulation of what are thought to be axonal-bound vesicles at the Golgi, similar to what is seen in Syt-IV knockdowns (Yonekawa et al., 1998; Arthur et al., 2010). Furthermore, Syt-IV knockout mice mirror learning and memory deficits similar to KIF1A knockout animals (Kondo et al., 2012). As such, HSP-related KIF1A mutants, whether loss-of-function or gain-of-function, may result in misdelivery of Syt-IV, thereby dysregulating synaptic vesicle fusion and release of a variety of neurotrophins, altering LTP, and leading to downstream effects such as learning and memory defects, problems with motor control, and other brain region specific effects.

### Tissue Plasminogen Activator (tPA) and Neuroserpin

DCVs transport secreted proteases and protease inhibitors such as tPA and neuroserpin (Lochner et al., 1998; Ishigami et al., 2007). tPA is a serine protease best known for its role in the conversion of the proenzyme plasminogen to active plasmin in the blood clotting pathway. It is also expressed widely throughout the nervous system in both neurons and glial cells, particularly within the cerebellum, hippocampus, and hypothalamus (Melchor and Strickland, 2005). tPA is involved in a variety of neuronal processes including synaptic plasticity, neuron migration, outgrowth, survival (Tsirka et al., 1995; Seeds et al., 1999; Samson and Medcalf, 2006; Chevilley et al., 2015). Notably, tPa/plasmin plays a role in processing of proBDNF to BDNF (Barnes and Thomas, 2008). This processing is critical for late-phase LTP in the hippocampus, a process underlying long-term memory. In affected patients, this may manifest as an intellectual disability. Additionally, tPA can promote NMDAR-mediated neurotoxicity, a mechanism implicated in a number of neurodegenerative disorders (Parcq et al., 2012; Spalloni et al., 2013; Liu et al., 2019). This may suggest that the control of tPA trafficking to synapses, along with control of tPA activity through inhibitors such as neuroserpin, is critical in mediating cell survival. Notably, mouse models of neurodegenerative disease express higher levels of tPA and other plasminogen activators, resulting in Wallerian and Wallerian-like degeneration (a “dying-back” phenotype in peripheral neurons and CNS neurons; Bignami et al., 1982; East et al., 2005). Furthermore, when these mouse models were crossed with transgenic mice that overexpressed neuroserpin, a greater number of motor neurons and myelinated axons were retained (Simonin et al., 2006). Relating this to KIF1A-mediated HSP, it is possible that there is a disequilibrium in the trafficking of either tPA or neuroserpin, ultimately leading to the aforementioned neurodegenerative phenotype. As these proteins are both highly expressed in regions related to learning, memory, seizure activity, and motor control, dysfunction in any of these areas could contribute to symptomology seen in complicated forms of SPG-30.

### Vesicular Monoamine Transporter 2

Dense core vesicles also traffic membrane proteins such as VMAT2, a confirmed KIF1A cargo (Liu et al., 2012). The integral membrane protein VMAT2 plays a crucial role in transport and accumulation of monoamines such as dopamine, norepinephrine, and serotonin into synaptic vesicles (Liu and Edwards, 1997; Yaffe et al., 2018). VMAT2 is concentrated in monoaminergic neuronal populations such as the nigrostriatal dopaminergic neurons (Schafer et al., 2013), playing an important role in packaging and release of dopamine and GABA. Mouse models with VMAT2 knockdown show a marked increase in nigrostriatal degeneration (Caudle et al., 2007), supporting VMAT2’s importance in the proper functioning and viability of this neuronal population. Nigrostriatal system involvement is sometimes seen in complicated forms of HSP (Kim et al., 2013) suggesting that VMAT2 may play a role. Possible dysfunction in VMAT2 trafficking, whether due to mislocalization or insufficient trafficking to synapses, may result in the aforementioned neurodegenerative phenotype, as well as nigrostriatal symptomology such as parkinsonism and other motor deficits.

### Netrin-1

Netrin-1 is a secreted protein that acts in axonal pathfinding and cortical and cerebellar migration (Leonardo et al., 1997; Boyer and Gupton, 2018). Whether netrin-1 is packaged within DCVs is currently unknown. However, UNC-104/KIF1A has been implicated in trafficking of its homologue UNC-6 in *C. elegans* (Ogura et al., 2012) and in rats with intracerebral hemorrhage, KIF1A abolishment led to attenuation of netrin-1-related functions (Wang et al., 2018). Notably, netrin-1 seems to be critical for guidance of CST axons, particularly within the pyramidal decussation and dorsal funiculus as netrin-1 mutant mice show defects in these regions (Finger et al., 2002). This may underlie the muscle weakness that is seen in pure and complicated HSPs. Moreover, netrin-1 also has a role in the modulation of APP signaling, as APP is a receptor of netrin-1 (Lourenço et al., 2009). In Alzheimer’s disease (AD) mouse models that have a decreased expression of netrin-1, an increase in Aβ levels is observed: a phenotype implicated in neurotoxicity (Lourenço et al., 2009). As netrin-1 is expressed in the hippocampus, it is possible that such effects may be underlying the cognitive symptoms seen in HSP.

### Tropomyosin Receptor Kinase A (TrkA)

Another essential neuronal cargo is the tropomyosin receptor kinase A (TrkA), a receptor with a high affinity for NGF (Tanaka et al., 2016). Upon binding of NGF, the TrkA-NGF complex is internalized and trafficked retrogradely to stimulate a PI3K-dependent signaling cascade that promotes neuronal survival (Marlin and Li, 2015; Tanaka et al., 2016). TrkA most likely does not have a critical role in pure forms of HSP as this receptor is not expressed within neurons of the CST, nor is NGF neuroprotective to CST injury (Giehl and Tetzlaff, 1996; Liebl et al., 2001). However, as TrkA is expressed extensively in the cholinergic system of the basal forebrain that contain extensions that innervate various regions that affect cognition, learning, and motor control, there may be a role of KIF1A-mediated TrkA trafficking dysfunction in some complicated forms of HSP (Sobreviela et al., 1994; Wu et al., 2014). It is important to note that to our knowledge, no studies have looked for basal forebrain cholinergic dysfunction in either form of HSP.

Instead, the high degree of TrkA expression within the dorsal root ganglion (DRG) primary afferent nociceptor sensory neurons may underlie the pathology seen in the KIF1A-dependent disorder HSAN II (Verge et al., 1992). Using KIF1A-haploinsufficient mouse models, Tanaka et al. demonstrated that mice not only developed sensory neuropathy with an ablated pain response similar to that seen in HSAN II patients, but using live imaging studies in DRG sensory neurons that TrkA trafficking to distal axons and sensory neuron survival was significantly reduced (Tanaka et al., 2016). The loss of sensory neuron survival is consistent with KIF1A-knockout mouse models, which also display decreased sensory neuronal survival (Yonekawa et al., 1998). Although known KIF1A-dependent HSAN II mutations in patients have not been characterized cellularly, they show a truncation at the cargo-binding PH domain that may copy the phenotype of the KIF1A-haploinsufficient mouse models (Riviere et al., 2011; Tanaka et al., 2016). As such, patients with such mutations may exhibit similar sensory loss resulting in ablated pain and other nociceptive losses. Interestingly, TrkA is also expressed within the superior cervical ganglion, a region involved in autonomic, particularly sympathetic nervous system responses, and is critical for its survival (Fagan et al., 1996). However, patients with KIF1A-induced HSANII show minimal to no autonomic disturbances (Riviere et al., 2011). This suggests that these neurons may not be particularly vulnerable to KIF1A-induced effects. It is interesting to note that in HSAN IV, or the congenital insensitivity to pain with anhidrosis (CIPA), the TrkA gene is mutated causing a loss of function of the receptor which results in attenuation of pain and temperature sensation similar to that of HSAN II (Indo, 2001). Inability to traffic TrkA should also mimic the loss-of-function phenotype seen in HSAN IV.

TrkA expression is also found in retinal ganglion cells (RGC) whose axons constitute the optic nerve (Bai et al., 2010). TrkA is integral for RGC survival as TrkA is upregulated in response to retinal and optic nerve damage, as well as the neuroprotectivity of a TrkA-selective NGF mutants in response to optic nerve and retinal damage (Bai et al., 2010; Mesentier-Louro et al., 2019). KIF1A mutations have been found in PEHO syndrome in which histological studies show a marked reduction in nerve fibers of the optic nerve and death of RGC layers (Somer et al., 1993; Esmaeeli-Nieh et al., 2015; Lee J.R. et al., 2015; Ohba et al., 2015; Samanta and Gokden, 2019). All of the mutations implicated in PEHO syndrome, such as the T99M mutation, are found within the motor domain of KIF1A, suggesting that motor motility is impaired. Indeed, two of the mutations, T99M and E253K, have been studied *in vitro*, with a proximal axonal and cell body distribution being seen along with a loss of motility (Esmaeeli-Nieh et al., 2015; Cheon et al., 2017). Although no studies to our knowledge have studied these mutants with respect to TrkA localization in RGC’s and their axons, we suspect that a similar phenotype to that seen in the Tanaka et al. study would be seen, i.e., failure of TrkA delivered to distal axonal sites.

### Autophagy-Related Protein 9

Autophagy is a general cellular mechanism required for protein clearance and recycling of damaged and aging organelles. In neurons, autophagy also plays a crucial role in a variety of developmental processes such as synapse development and neurite outgrowth (Shen et al., 2015). Disruption to the autophagic process is a central contributor to a host of neurodegenerative diseases. The autophagosome is the functional unit of autophagy and requires ATG-9, the only transmembrane protein of the ATG family involved in its formation. Though ATG-9A is ubiquitously expressed in mammalian cells, it is highly enriched in neurons (Tamura et al., 2010). It is typically found at autophagic sites in the axon terminal, however, is also distributed throughout the somatodendrites and axons, and is thought to play an integral role in sequestering membrane from organelle donors for autophagosome formation (Tamura et al., 2010; Feng and Klionsky, 2017). A study found that in *C. elegans* KIF1A/Unc-104 transports ATG-9 to neurite tips and was critical for cytoskeletal organization and synaptic architecture (Stavoe et al., 2016). In vertebrates, ATG-9A deficient neurons show reduced neurite outgrowth, and in ATG-9A knockout mice signs of neurodegeneration are seen, including spongiosis of nerve fibers in both axonal terminals and axons (Yamaguchi et al., 2018). Failure to deliver ATG-9A to distal axons via a KIF1A mutant might show similar effects. In AP-4 deficiency syndrome, another form of complicated HSP, there is mislocalization of ATG-9A from the peripheral cytoplasm to the *trans*-Golgi, which results in symptomology akin to that seen in KIF1A-mediated complicated HSP (De Pace et al., 2018). Currently, no studies have been performed in vertebrate neurons to assess KIF1-ATG-9A trafficking.

### β-Secretase 1 (BACE1)

β-Secretase (BACE1), a KIF1A cargo, is an aspartyl protease that regulates critical processes including neuronal growth, function, repair, and myelination (Hung and Coleman, 2016; Yan, 2017) by processing a number of essential proteins. One notable substrate of BACE1 is the APP, where BACE1 cleavage leads to formation of amyloid beta (Aβ) (Puzzo et al., 2008). APP can be cleaved at multiple locations within the cell, for example in the ER, along the trafficking pathway, and at the plasma membrane. Perturbing APP transport at any step can lead to misprocessing. A result of this might be a reduction in APP required for functions such as neurite outgrowth and synaptogenesis. Additionally, this can lead to generation of excessive Aβ, which is toxic. Though more typically associated with AD, Aβ plays a physiological role in neurons. For example, picomolar concentrations of Aβ 1–42 monomers and oligomers are needed to increase hippocampal LTP and episodic memory formation (Puzzo et al., 2008, 2011; Abramov et al., 2009). KIF1A mutations that mislocalize BACE1 may cause a shift in the balance between physiological APP and Aβ, leading to changes in nervous system development, plasticity and cognition, thus contributing to HSP pathology.

In addition to mislocalization, certain KIF1A mutants (i.e., V8M, A255V) may lead to an over-accumulation of BACE1 at APP rich sites leading to overproduction of Aβ. APP can function as an unconventional G-protein coupled receptor linked to the G_o_ signaling pathway that contributes to neuronal outgrowth and survival (Nishimoto et al., 1993; Milosch et al., 2014; Copenhaver and Kögel, 2017). Aβ promotes APP multimerization at cell-surface sites leading to activation of the heterotrimeric G_o_ protein. Dysregulated APP-G_o_ signaling promotes cellular toxicity in AD eventually resulting in neuronal degeneration (Copenhaver and Kögel, 2017). Moreover, increased processing of APP may also produce Aβ peptides that act as synaptic toxins analogous to the effects found in AD. A similar mechanism may be at play in KIF1A-related HSP. Furthermore, NMJ synaptic maturation in insect models is dependent on APP-G_o_ signaling (Luchtenborg et al., 2014). Thus, altered Aβ production may perturb the homeostatic G_o_ signaling in this developmental process. Although this should be replicated in mammalian models, dysregulation of NMJ synapse maturation may be a possible pathological mechanism caused by KIF1A mutants and could explain certain symptoms such as the progressive muscular weakness seen in HSP.

BACE1 also cleaves the cell adhesion molecule NRG1 (Willem, 2016). NRG1 is critical in regulating PNS myelination, development of Schwann cells, and development of muscle spindles (Fleck et al., 2012). Cleaved NRG1 forms a complex with ErbB, which subsequently binds onto Schwann cells, activating downstream phosphorylation of Akt leading to myelination of the axon. HSP-related KIF1A mutants may result in BACE-1 mislocalization thereby impairing Schwann cell myelination of peripheral nerves. As such, these BACE1 detriments likely contribute to KIF1A spasticity and the motoneuron axon atrophy seen in peripheral neuropathy. Notably, Fleck et al. (2012) demonstrated both BACE1 null neurons and heterozygous mutant NRG1 expressing neurons resulted in hypomyelinated peripheral neuron axons, indicating that deficiencies in either protein’s function results in the same phenotype. Furthermore, BACE1-NRG1 signaling also controls the development and maintenance of muscle spindles (Klebe et al., 2012) with BACE1 knockout mice having malformed and underdeveloped muscle spindles (Cheret et al., 2013). Because proper muscle spindle function is essential for muscle lengthening and subsequent correct muscle reflexes (Chen et al., 2003), BACE1 mislocalization may contribute to the hyperreflexia seen in SPG-30.

SPG-30 and PEHO patients may also exhibit optic nerve impairments. Notably, BACE1 null mice display oligodendrocyte-mediated hypomyelination and decreased axonal regeneration in the optic nerve (Hu et al., 2006), which may be caused by KIF1A-BACE1 localization defects. In the retina, BACE1 cleaves Vascular Endothelial Growth Factor (VEGF1) to inhibit lipofuscin deposition and retinal thinning (Cai et al., 2012). Mislocalization of BACE1 due to KIF1A mutation may explain the impairments mentioned above. Further studies must be done utilizing histopathological data of KIF1A-mutant HSP patients, or using KIF1A-mutant mouse models to confirm the cause of these retinal defects.

Finally, patients with KIF1A mutations also present with epilepsy, where some forms are connected to altered voltage-gated ion channels activity. BACE1 is involved in the proteolytic processing of the sodium channel subunit Na_v_β_2_ at the plasma membrane (Kim et al., 2007, 2011). When Na_v_β_2_ remains uncleaved by BACE1, there is a reduction in Na_v_1.1a at the plasma membrane (Kim et al., 2011). As a result, neurons compensate by upregulating a different sodium channel, Na_v_1.2. This upregulation is observed in BACE1 knockout neurons as hyperexcitability (Hu et al., 2010). The interaction of BACE1 and Na_v_β_2_ subunits occurs at the plasma membrane, thus KIF1A-BACE1 transport defects and decreased BACE1- Na_v_β_2_ processing may account for changes in sodium channel levels and composition that contribute to HSP epilepsy.

## Kif1C Structure and Function

**FIGURE 3:**
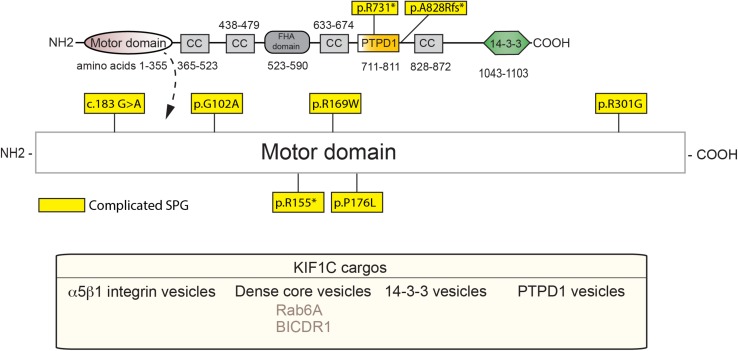
Schematic diagram of KIF1C and HSP mutations (adapted from Siddiqui et al., 2019; UniProt #043896). See Supplementary Table 2 for details of each mutation. Below, KIF1C cargos.

KIF1C, also a kinesin-3 family member, is causal in HSP (SPG58). KIF1C is a ubiquitously expressed motor that is involved in retrograde Golgi to ER transport, as well as anterograde transport of α5β1 integrin and DCVs (Dorner et al., 1998; Schlager et al., 2010; Theisen et al., 2012). KIF1C also acts in the maintenance of Golgi structure (Simpson et al., 2012; Lee P.L. et al., 2015). As with the rest of the kinesin-3 family members, KIF1C belongs to the Walker-type ATPase family, which constitutes a P-loop and Walker A and B motifs, as defined previously. The motor domain sequence is highly conserved, with the sequence similarity between KIF1C and KIF1A being around 81% (Lee P.L. et al., 2015). Dorner et al. (1999) showed that KIF1C may form four coiled-coil domains in the region of amino acids 370, 450, 640, and 850. The stalk and the tail domains, however, differ as compared to KIF1A with the tail being homologous to KIF1Bα. Within the C-terminus, a PTPD1-binding domain (amino acids 714–809) has been defined (Dorner et al., 1998), along with a CBD that bind Rab6A and 14-3-3 family members (Figure 3; Dorner et al., 1999; Lee P.L. et al., 2015).

KIF1C regulation is complex and not well defined, with both inherent autoinhibitory components as well as cargo-mediated regulation. KIF1C, unlike the autoinhibited monomers of KIF1A, exists as an autoinhibited dimer *in vivo* (Siddiqui et al., 2019). Recently, Siddiqui et al. (2019) demonstrated that autoinhibition occurs between the stalk domain and the motor domain of KIF1C. More specifically, the FHA domain, the third coiled-coil domain, and the microtubule-interacting region of the motor domain associate resulting in autoinhibition of the KIF1C dimer by preventing its binding to MT. Certain mechanisms regarding cargo-mediated regulation of KIF1C and how it relates to motor function, whether that be changes in microtubule-motor interactions, motor autoinhibition, directionality, or cargo binding, have been elucidated. One of the first known interactors of KIF1C, the protein-tyrosine phosphatase PTPD1, has recently been implicated in activation of KIF1C. Siddiqui et al. (2019) demonstrated that the FERM domain of PTPD1 is capable of binding the stalk domain of KIF1C to relieve the autoinhibition between the dimers, independently of its catalytic or phosphatase activity. Furthermore, Siddiqui et al. (2019) showed that Hook3, a microtubule-binding protein that associates with KIF1C, bound to a region close to that of PTPD1 and also relieved autoinhibition. Another known KIF1C interacting partner is the BICD family like cargo adaptor 1 (BICDR1) which is thought to act as a trafficking regulator. Low levels of BICDR1 result in localization of the GTPase-Rab6 vesicles, a cargo of KIF1C, to cytosolic regions within cells, while high levels result in retention of these vesicles in pericentrosomal regions (Schlager et al., 2010). As BICDR-1 also interacts with the dynein-dynactin complex, BICDR-1 may act as a directionality switch for Rab6/KIF1C complexes (Urnavicius et al., 2018). Hook3 also interacts with the dynein-dynactin complex, suggesting a similar switching role to that of BICDR-1 (Urnavicius et al., 2018). A particularly interesting regulator of KIF1C is Rab6, as it is also a bona fide cargo of this molecular motor (Lee P.L. et al., 2015). In addition to interacting with the CBD of KIF1C, Rab6 can bind to the motor domain directly (Lee P.L. et al., 2015). This interaction blocks KIF1C microtubule binding, particularly when KIF1C is in an ATP-On (microtubule binding) state. Kinases have also been implicated in the regulation of KIF1C cargo-binding. In order to bind 14-3-3 to KIF1C, the serine at position 1092 must be phosphorylated via casein kinase II (CKII) (Dorner et al., 1999). Phosphatase activity may also directly affect motor function, with the possibility that PTPD1, in addition to its role as an activator of KIF1C, may also modulate the motor in a phosphatase-dependent manner. Taken together, mutations that affect any one of the various motor, cargo-binding or regulatory domains have the potential to dysregulate KIF1C-mediated activity.

## Kif1C Mutations in Spg-58 and Related Disorders

To our knowledge, all known SPG-58 causing KIF1C mutations are familial and can be either recessive or dominant. Furthermore, they present clinically as a complicated form of HSP, although other KIF1C-related disorders are seen. These mutations are found almost exclusively within the motor domain with two other deletion mutants: one which truncates the stalk domain, and one that leads to degradation by nonsense-mediated decay (Figure 3 and Supplementary Table 2). For example, the R301G mutant, which is found within the highly conserved loop 12, may affect microtubule binding while the A828Rfs^∗^13 mutation, may truncate the fourth coiled-coil and CBD which would most likely ablate binding of cargos such as Rab6 and the 14-3-3 proteins (Yücel-Yılmaz et al., 2018). As with KIF1A mutants, the extent and severity of SPG-58 symptomology is dictated by the changes in KIF1C amino acid residues and the patient’s overall genetic background.

Most of the known SPG58 causing mutations have not been characterized, however, Oteyza et al. (2014) defined three motor mutants that are causal in SPG58 patients. Two of these mutants, G102A and R301G, which affect the Walker A motif involved in nucleotide binding and loop 12 involved in microtubule binding, respectively, accumulate perinuclearly as opposed to the normal accumulation at the cell periphery. By contrast the P176L mutant accumulated peripherally similar to endogenous KIF1C. Motility studies are needed to confirm if this is either loss or gain-of-function. Another mutation at G102A appeared to reduce KIF1C stability thereby reducing overall KIF1C levels. In a previous study, Dorner et al. (1998) introduced a lysine to alanine mutation in an adjacent amino acid, K103, which halted Golgi to ER trafficking, suggesting mutations in this region are critical for KIF1C motility. Both the position of the mutation and the nature of the amino acid change appear to be critical in determining the extent of impairment that may be seen in the motor. Furthermore, Oteyza et al. (2014) found that parents of patients carrying the mutations were heterozygous for those alleles and demonstrated a subclinical phenotype, as opposed to the severe presentation seen in patients that were homozygous for the mutant allele. This indicates that mutant homozygosity is required for full presentation of SPG58 as all the known mutations that result in full presentation in SPG58 patients have been homozygous to date. Co-ordinate expression of the G102A mutant, along with wild-type KIF1C, showed a normal localization pattern. This suggests that in heterozygous patients, partial KIF1C function is retained, ameliorating disease severity. Confounding the issue are studies with KIF1C-null mice that have normal embryonic development, fertility, and viability (Nakajima et al., 2002). This implies there are functionally compensatory kinesins, and that the presence of mutations may interfere with cellular processes more drastically than a complete loss of KIF1C.

## Kif1C Cargos

**FIGURE 4:**
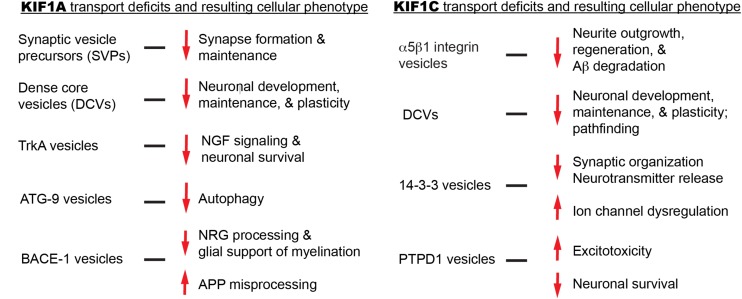
Summary of KIF1A and KIF1C cargos and the potential cellular abnormalities resulting from trafficking defects.

We will explore several KIF1C cargos in an attempt to bridge motor mutations, transport aberrations, and clinical symptomology (Figure 4). Where possible, we will also attempt to focus on the impact of cargo mislocalization in specific neuronal subtypes, for example, differences between CNS and PNS neurons.

### Rab6-BICDR1

The small GTPase-Rab6 is a known marker of secretory vesicles and is thought to control spatial distribution of exocytosis within cells (Grigoriev et al., 2007). Rab6 family members include the ubiquitously expressed Rab6A, and Rab6A’, as well as Rab6B, which is enriched in the brain (Echard et al., 2000; Wanschers et al., 2007). Rab6A, similarly to other members of the family, interacts with the CBD of KIF1C, as well as with loops 6 and 10 of the motor domain: the latter acting as a microtubule binding inhibitor (Lee P.L. et al., 2015). Furthermore, Rab6 interacts with BICDR-1, a regulator of KIF1C movement, with the strongest interaction occurring between the brain-enriched Rab6B and BICDR-1 (Schlager et al., 2010). Additionally, Rab6 co-localizes with DCV markers such as NPY, BDNF, and Sema3a in hippocampal neurons (Schlager et al., 2010). As Rab6-secretory vesicles traffic to distal neuronal processes, many of the same concepts regarding the cargos NPY and BDNF that were discussed in the KIF1A DCV section apply.

An interesting avenue to explore would be whether HSP-causing mutations affect KIF1C-BICDR-1 or KIF1C-Rab6 interactions leading to changes in intracellular distribution of the DCV cargos, and the overarching effects on neuronal populations implicated in SPG58. Indeed, changes in BICDR-1 expression result in changes in secretory vesicle localization, as does Rab6A (Schlager et al., 2010; Lee P.L. et al., 2015). Furthermore, knockdown of Rab6 and sustained expression of BICDR-1 do exert negative changes, particularly decreases in neurite outgrowth (Schlager et al., 2010). In certain SPG58 cases, embryonic and early postnatal development is normal, indicating that neuronal migration is complete at an early stage (Dor et al., 2014). This implies that potential BICDR-1/Rab6 pathfinding defects may begin late in post-natal development. As both Rab6 and BICDR-1 have binding sites at the C-terminus of KIF1C, it follows that mutations in these regions could result in cargo binding deficits. However, as only two known mutants, R731^∗^ and A828Rfs^∗^13, affect these regions, with the former resulting in nonsense-mediated decay, this could not explain all the other cases of SPG58 (Dor et al., 2014; Yücel-Yılmaz et al., 2018). It is possible, however, that mutations within the motor domain may cause steric changes that disallow direct interactions between Rab6A and KIF1C, thereby relieving the Rab6A-dependent microtubule-binding inhibition. This may result in aberrant trafficking of KIF1C cargos resulting in a variety of changes to neurite growth, neuronal development, and survival in a cargo-dependent manner.

### Semaphorin 3a (Sema3a)

Sema3a is a membrane-associated secreted protein that has roles in axon guidance, growth, and neuronal migration (Schwamborn et al., 2004; Kruger et al., 2005; de Wit et al., 2006). It is widely expressed throughout the CNS and functions as a guidance cue that can act as an axonal chemorepellent, or as a chemoattractive agent for cortical apical dendrites (Polleux et al., 2000; Vo et al., 2013). Notably, in corticospinal neurons, ventral spinal cord-derived Sema3A interacts with a neuropilin-1/L1CAM complex that is subsequently internalized. This results in a shift away of the corticospinal axons from the medullary junction (Bechara et al., 2008; Blackstone, 2012), highlighting possible neurodevelopmental defects in CST formation in HSP. It is possible that the inability to ferry this cargo to sites of release during the formation of the CST may result in neuronal guidance defects. In a separate Sema3A-related mechanism, internalization of Sema3A in hippocampal and subicular pyramidal neurons lead to neurodegeneration in an AD model (Good et al., 2004). Furthermore, in the motor cortex of patients affected with ALS, Sema3A expression is highly upregulated, suggesting that it is involved in the degeneration of motor neurons (Korner et al., 2016). These two mechanisms would seem to oppose each other in KIF1C-related HSPs as failure to deliver Sema3A to sites of release should lead to CST defects, but should be neuroprotective, or at least not induce degeneration, of neurons within the hippocampus/subicular pyramidal neurons or that of the motor cortex. It may be that depending on the mutation in KIF1C, whether it is loss-of-function or gain-of-function, may result in different HSP pathophysiology. Inability to deliver Sema3A to sites of release may result in greater CST defects, whilst more hippocampal/motor cortex defects may arise in gain-of-function mutations that cause increased delivery of Sema3A.

### α5β1 Integrin

α5β1 integrin, also known as the fibronectin receptor, is a transmembrane heterodimer that is trafficked by KIF1C. Multiple thorough reviews exist that summarize the functions of α5β1 integrins in neurons (Clegg et al., 2003; Wu and Reddy, 2012; Lilja and Ivaska, 2018), therefore, we will only focus on a select few that may influence HSP pathology. α5β1 is expressed throughout the CNS and PNS including regions such as the hippocampus, cerebellum, and cerebral cortex (Wu and Reddy, 2012). Expression varies throughout development, as developing neurons express α5β1 in higher levels than that of mature neurons (Wu and Reddy, 2012). α5β1 is important for a variety of neuronal processes including neurite outgrowth, synaptogenesis, and more broadly, plasticity (Tonge et al., 2012; Wu and Reddy, 2012; Nishimura et al., 2016). More specifically, α5β1 subunits play roles in synaptic cytoskeletal organization, LTP, and hippocampal presynaptic release probabilities (Huang et al., 2006; Webb et al., 2007; Babayan et al., 2012; Lilja and Ivaska, 2018). In mice with excitatory neuron-specific β1 integrin knockouts, NDMAR-dependent LTP and AMPA receptor-mediated synaptic transmission were impaired and as a consequence; working memory (Chan et al., 2006). As such, intellectual disabilities seen in SPG58 may possibly be explained by such mechanisms. Although this study focused mainly on hippocampal regions, the CST neurons are mainly excitatory glutamatergic neurons, perhaps indicating that similar synaptic defects may be at play underlying the weakness seen in SPG58. Furthermore, it is possible that neuronal outgrowth and regeneration in response to neuronal damage may be impaired in a α5β1-dependent manner. Both expression of α5 and β1 subunits is sufficient to enhance neurite outgrowth and regeneration (Condic, 2001; Clegg et al., 2003). Insufficient or ablated trafficking of these subunits by KIF1C may result in various neurodevelopmental defects, and possibly reduced regeneration in peripheral neurons that are damaged by SPG58-related pathogeneses. In another example, α5β1 integrin protects against Aβ deposition, where Matter et al. demonstrated that α5β1-expressing cells showed increased degradation of Aβ 1-40, reducing Aβ-induced apoptosis (Matter et al., 1998). As possible Aβ-mediated neurodegeneration has been a recurring theme in our exploration of kinesin-3 cargo function, this would be an exciting avenue to explore regarding HSP pathogenesis.

### 14-3-3

Yeast-two hybrid screens have shown an interaction between the 14-3-3 family members β, γ, ε, ζ and KIF1C, with coimmunoprecipitation studies confirming an interaction between the γ-isoform and KIF1C (Dorner et al., 1999). The 14-3-3 family of proteins are a group of ubiquitously expressed regulatory proteins that are particularly enriched in the brain, constituting approximately 1% of total soluble proteins (Berg et al., 2003). 14-3-3 protein functions include acting as an intracellular scaffold, as well as with binding to target proteins thereby masking their localization signals and regulatory sites (Cornell and Toyo-oka, 2017). 14-3-3 proteins function in neurite outgrowth, synaptogenesis, ion channel regulation, and release of neurotransmitter. Furthermore, 14-3-3 proteins are implicated in a variety of neurodegenerative disorders including Alzheimer’s and Huntington’s disease, ALS, and spinocerebellar ataxia type 1. Many thorough reviews exist detailing the function of 14-3-3 family members in neurons and in neurological disorders (Berg et al., 2003; Foote and Zhou, 2012; Cornell and Toyo-oka, 2017; Zhang and Zhou, 2018). However, no studies to our knowledge explore 14-3-3 proteins in HSPs.

A few interesting avenues exist to explore 14-3-3 protein-KIF1C interactions in HSP. The distribution of 14-3-3 protein isoforms differ between different regions of neurons. For example, γ and ε –isoforms are found at presynaptic sites and the ζ isoform is found on synaptic vesicles (Martin et al., 1994; Broadie et al., 1997). Potential studies should explore the possibility of KIF1C-mediated transport of 14-3-3 proteins. The inability of 14-3-3 isoforms such as γ and ε to reach presynaptic sites could have a variety of consequences on overall synaptic function. One such consequence may be dysregulation of Ca_V_2.2 ion channels critical for mediating Ca^2+^ influx for neurotransmitter release. Electrophysiological studies demonstrated that 14-3-3 γ and ε inhibition results in increased inactivation kinetics of Ca_V_2.2 channels, with a corresponding change in short-term synaptic plasticity (Li et al., 2006), which may alter neural circuits which underlie the spasticity seen in HSP. Furthermore, the ζ-isoform seems to play a role in glutamatergic synapse formation and neuronal navigation (Cheah et al., 2012). Cheah et al. generated 14-3-3 ζ knockout mice and found that they exhibit hippocampal abnormalities such as abnormal mossy fiber navigation (Cheah et al., 2012; Xu et al., 2015). Symptomology similar to that of certain KIF1C-mediated complicated HSP such as learning and memory defects were seen in these mice. It is important to note that no motor defects were seen, suggesting that the ζ-isoform does not participate in CST glutamatergic synapse formation, or there is possibly redundancy between 14-3-3 proteins.

### PTPD1

The protein tyrosine phosphatase PTPD1 was primarily identified as a KIF1C interactor, yet more recently was described as a regulator of KIF1C autoinhibition with Siddiqui et al. (2019) postulating that it may also be a cargo (Dorner et al., 1998). PTPD1 interacting partners play a number of roles in neuroprotective and neurodegenerative pathways. For example, Src tyrosine kinase, when in complex with PTPD1, is activated, and that rapid activation of Src is integral in glutamate-induced excitotoxic neurodegeneration (Cardone et al., 2004; Khanna et al., 2007). Using a Src knockdown approach, they demonstrated that glutamate-induced neurodegeneration was decreased. If PTPD1 is indeed a cargo, gain-of-function KIF1C mutants may result in over accumulation of PTPD1 at Src sites sensitizing excitatory neurons to glutamate, resulting in a neurodegenerative cascade. Another interacting partner of PTPD1 is the EGFR, in which PTPD1 acts as a potentiator of EGFR activity (Roda-Navarro and Bastiaens, 2014). This potentiation is dependent on recruitment of PTPD1 to EGFR sites, which could relate to its possible dependence on trafficking by KIF1C. EGFR is implicated in cortical neuronal survival via an astrocyte-mediated mechanism (Wagner et al., 2006). Knockout of EGFR in cortical astrocytes resulted in an Akt-dependent apoptotic cascade. It is possibly that a similar phenotype may be seen if PTPD1 was unable to be recruited to EGFR sites to allow for potentiation and subsequent proper activation. This may have widespread effects throughout the cortex, possibly affecting a variety of neuronal populations that are associated with HSP symptomology, such as hippocampal and upper motor neurons.

## Selective Neuronal Vulnerability in Hsp

A widespread and unsolved issue in neurodegeneration is understanding the underlying cause of selective neuronal vulnerability (Han et al., 2010; Fu et al., 2018). In both pure and complicated forms of HSP, a heterogeneous population of neurons constituting different regions of the CNS and PNS are affected. This raises questions of why these specific tissues and nerves are involved, why different mutants may affect different subsets of nerves, as well as how the same mutation may affect different neuronal populations in different patients. Understanding the exact neuronal populations that display HSP-related pathology is critical to understanding the specific properties that render them selectively vulnerable. Currently, the neuroanatomy is well characterized in HSP; however, information regarding specific affected neuronal populations is incomplete (Bruyn, 1992; Fink, 2013). One proposed idea is that axon length confers a particular vulnerability to degeneration of affected neuronal populations such as the CST and *fasciculus gracilis* nerve fibers (Han et al., 2010; Fink, 2013). However, this concept has been challenged as certain regions affected do not constitute neurons with long axons, such as that of the cerebellum and basal ganglia (Han et al., 2010; Fink, 2013). It may not therefore encompass the entirety of the vulnerability but may well be a compounding factor. The genetic background of the patient also plays a role in the differing phenotypic presentation between mutants. This concept has been reviewed at length with factors such as epigenetic changes, stochasticity, penetrance, and expressivity all playing a role in determining HSP phenotypes (Cooper et al., 2013; Kammenga, 2017).

Other factors that may contribute to neuron-specific vulnerability may be motor redundancy, or the ability of one motor type to compensate for another. Motors such as KIF5, KIF1C and KIF1A may have overlapping or redundant roles in the transport of BDNF- and NPY-containing vesicles (Gauthier et al., 2004; Lim et al., 2017; Stucchi et al., 2018). Furthermore, there may be motor isoform differences between neuronal populations which contribute to their vulnerability. KIF1A, for example, has 3 defined isoforms, with 13 other potential isoforms (The Uniprot Consortium, 2019). As such, these different isoforms may display varying functions in different neuronal populations, with mutations detrimentally affecting one specific isoform more so than another. Moreover, cytoskeletal differences between neuronal populations may contribute to their vulnerability. For example, tubulin isoforms vary greatly between different regions in the CNS and within different neurons of the same regions (Bai et al., 2008; Tischfield and Engle, 2010; Hawrylycz et al., 2012). Notably, motor dynamics are altered via differences in tubulin isotypes, as well as differences in post-translational modifications (Sirajuddin et al., 2014; Lessard et al., 2019). Polyglutamylation of tubulin, for example, results in dynamic changes in both KIF5 and KIF1A motility. For KIF5, polyglutamylated tubulin results in an increase in motility, while KIF1A run lengths and pause durations are decreased; processes that are critical to maintaining KIF1A’s velocity (Sirajuddin et al., 2014; Lessard et al., 2019). Mutants may display differential effects depending on which tubulin isotype is expressed, and the post-translational modifications present within specific neuronal sub-populations.

Lastly, preferential susceptibility to apoptotic signaling cascades, particularly those conferred via glutamate-induced excitotoxicity, has been a recurring theme throughout this review. To our knowledge, no studies have probed whether glutamate-induced excitotoxicity plays a role in HSP pathology. However, neuronal populations implicated in HSP have shown selective vulnerability to this mechanism. Susceptibility to glutamate-induced excitotoxicity is seen in certain neuronal populations associated with neurodegenerative disorders such as ALS and AD (Mattson et al., 1989; Lorenzo et al., 2006). For example, in ALS, vulnerable motor neurons display consistently low expression of inhibitory GABA and glycine receptors resulting in the neuron’s susceptibility for hyperexcitation (Lorenzo et al., 2006). Furthermore, these vulnerable populations of neurons display low levels of Ca^2+^ buffering proteins, which would contribute to the inability to resolve abnormal changes in intracellular Ca^2+^ levels (Vanselow and Keller, 2000). In ALS mouse models corticospinal motor neurons show selective, marked degeneration of apical dendrites in regions with high levels of cortical modulatory input (Jara et al., 2012). Notably, the mouse model of Alsin-mediated HSP showed a similar dendritic phenotype, with additional axonal pathology and mitochondrial and Golgi apparatus defects; organelles critical for intracellular Ca^2+^ homeostasis (Wong et al., 2013; Gautam et al., 2016; Bagur and Hajnóczky, 2017). In hippocampal neuron populations that are affected in AD such as the CA1 and CA3 neurons, vulnerability to glutamate-induced excitotoxicity is seen while resistance in other populations such as the dentate granule cells and CA2 pyramidal neurons (Mattson et al., 1989) is retained. It is important to note that other neurodegenerative pathways likely exist that we have not considered here including changes in protein homeostasis, mitochondrial energy demand, and differences in the synthesis of neurotransmitters and their cognate receptors (Fu et al., 2018).

## Future Directions

As with many other neurodegenerative disorders, HSP is neither treatable nor curable with current therapies. Rather, treatments center on relieving symptomology, such as the use of intrathecal Baclofen, and botulinum toxin injections to reduce spasticity, along with physical therapy for general motility and maintenance of strength (Fink, 2013; Bellofatto et al., 2019). To target the underlying pathophysiology in kinesin-3 motor-mediated HSP, therapeutics would have to be mutation specific, as mutants can exhibit either loss-of-function or hyperactivity. In KIF1A-mediated HSP, targeting of the motor itself is a good course of action as the motor is neuron-enriched with little to no expression in other tissues, thus minimizing off-target effects. Identifying or designing drugs that are unique to specific sequences within the KIF1A motor domain is a possible therapeutic route. However, due to the high sequence similarity in the motor domain of the kinesins, this could be challenging, but not without precedence. In the mitotic motor Eg5, for example, the cell-permeable small molecule inhibitor monastrol binds a specific sequence, leading to allosteric inhibition of ATPase activity and reduced MT-binding ability (Maliga et al., 2002). Therapeutics that target regulatory domains may also be desirable, such as identifying macrocycle scaffolds which force dimerization and would subsequently activate KIF1A (Ito et al., 2015). This approach would be particularly useful in cases such as the loss-of-function T99M mutant, where transport deficiencies may be overcome by activating additional motors on the cargo membrane. In hyperactive KIF1A mutants such as the V8M mutant, compounds that partially reduce motility would be advantageous. For example, small molecules or biologics that reduce the ATPase function, destabilize MT-KIF1A interactions, or reduce force generation, may lead to a greater chance of cargo delivery. Therapeutic design for KIF1C-mediated HSP may be more problematic as KIF1C is ubiquitously expressed, thereby targeting the motor may result in off-target effects. Although KIF1C is ubiquitously expressed, mutants tend to result only in neurological symptomology, suggesting that neurons are more susceptible to KIF1C disruption than other cell types. It may therefore be feasible to target the motor itself in ways similar to that of KIF1A without concurrent effects on non-target populations.

The Chinese philosopher Confucius once said that “going too far is the same as falling short.” This piece of wisdom is fully embodied by the kinesin-3 family members KIF1A and KIF1C in HSP and the related disorders HSAN IIC and PEHO syndrome. Both loss-of-function and gain-of-function motor mutants may result in aberrant subcellular localizations of essential neuronal cargos (Figure 4). These cargos play a variety of roles in the development, maturation, and viability of the nervous system, and many are implicated in other neurodegenerative disorders. To further our understanding of kinesin-3 motors roles in these disorders, cellular characterization of disease-related mutants needs to be carried out. Future studies should include characterization of motility properties, as well as changes in subcellular localization of the motors, and particularly their cargos. HSP pathology samples would yield clues regarding cargo mislocalization. To the best of our knowledge, such samples are limited, therefore experiments in mouse models and engineered neural stem cells will have to be designed to provide relevant data regarding kinesin-3 mediated pathophysiology. Ultimately, the goal of this review is to encourage future academic and clinical efforts to focus on “transportopathies” such as HSP through a cargo-centric lens.

## Author Contributions

DG and VL reviewed the literature, and wrote and edited the first draft of the manuscript. MS and SN conceived the idea and edited the final manuscript.

## Conflict of Interest Statement

The authors declare that the research was conducted in the absence of any commercial or financial relationships that could be construed as a potential conflict of interest.

## Abbreviations

Akt: protein kinase B
APP: amyloid precursor protein
AP-4: adaptor protein 4
ATG-9: autophagy-related protein 9
BACE1: beta-secretase 1
BDNF: brain-derived neurotrophic factor
BICDR1, bicaudal D-related protein 1;CaMKII: Ca^2+^/calmodulin-dependent protein kinase II
CBD: C-terminal binding domain
CC: coiled-coil
CNS: central nervous system
CST: corticospinal tract
DCV: dense core vesicle
DENN/MADD: differentially expressed in normal versus neoplastic/mitogen-activated protein kinase-activating death domain
EGFR: epidermal growth factor receptor
FHA: forkhead-associated domain
HSAN: hereditary sensory and autonomic neuropathy
HSP: hereditary spastic paraplegia
KIF: kinesin superfamily proteins
LTP: long-term potentiation
MT: microtubules
NC: neck coil
NGF: nerve growth factor
NL: neck linker
NMDAR: *N*-methyl -D-aspartate receptor
NMJ: neuromuscular junction
NPY: neuropeptide Y
NRG1: neuregulin-1
NT-3: neurotrophin-3
PEHO, progressive encephalopathy with edema: hypsarrhythmia and optic atrophy
PH: pleckstrin-homology
PNS: peripheral nervous system
PTPD1: protein tyrosine phosphatase D1
Sema3a: semaphorin 3a
SNARE: synaptosomal nerve-associated protein receptor
SPG: spastic paraplegia
SVP: synaptic vesicle precursor
SV2A: synaptic vesicle protein 2A
Syt-I/IV: synaptotagmin 1/4
TANC2, tetratricopeptide repeat: ankyrin repeat and coiled-coil containing 2
tPa: tissue plasminogen activator
Trk: tropomyosin receptor kinase
VMAT2: vesicular monoamine transporter 2
VAMP2: vesicle-associated membrane protein.
