# Supplementary material for: Going Too Far Is the Same as Falling Short†: Kinesin-3 Family Members in Hereditary Spastic Paraplegia
Source: Front Cell Neurosci. 2019 Sep 26;13:419. doi: 10.3389/fncel.2019.00419 (PMC6775250; doi:10.3389/fncel.2019.00419)
Supplement: Supplementary file 1 [file Table_1.docx]

**TABLE 1**

Motor Domain Mutations in KIF1A: Pure SPG-30, Complicated SPG-30, and PEHO Syndrome.

| Neurodegenerative  Condition | | Nature of Mutation | | Amino Acid Mutation (Nucleotide) | | Other Associated Neurological Conditions^$^ | | References |
| --- | --- | --- | --- | --- | --- | --- | --- | --- |
| **Pure SPG-30** |  | |  | |  | |  | |
|  | Dominant, familial | | p.V8M (c.22G>A) | | - | | Iqbal et al., 2017 | |
|  |  | | p.I27T (c.80T>C) | | - | | Iqbal et al., 2017 | |
|  |  | | p.S69L (c.206C>T) | | - | | Ylikallio et al., 2015;  Roda et al., 2017 | |
|  |  | | p.Y74C (c.221A>G) | | - | | Warrenburg et al., 2016 | |
|  | Recessive, familial | | p.A255V  (c.765C>T) | | - | | Erlich et al., 2011 | |
| **Complicated SPG-30** | |  | |  | |  | |  |
|  | | Dominant, de novo | | p.S58L  (c.173C>T) | | - Ataxia  - Cerebellar Atrophy  - Peripheral Neuropathy  - MRD9 | | Lee J.R. et al., 2015 |
|  | |  | | p.A85D  (c.254C>A) | | - Cerebellar Atrophy  -Epilepsy  -MRD9 | | Yoshikawa et al., 2019 |
|  | |  | |  | |  | |  |
|  | |  | | p.T99M  (c.296C>T) | | - Ataxia  - Cerebellar Atrophy  - Corpus Callosum Hypoplasia  - Cortical Visual Impairment  - Optic Nerve Atrophy  - Cerebral Atrophy  - Epilepsy  - MRD9  - Peripheral Neuropathy  - Microcephaly | | Hamdan et al., 2011; Okamoto et al., 2014;  Lee J.R. et al., 2015  Esmaeeli-Nieh et al., 2015 |
| **Complicated SPG-30, cont.** | |  | | p.G102S  (c.304G>A) | | - Peripheral Neuropathy | | Citterio et al., 2015 |
|  | |  | | p.G102D  (c.305G>A) | | - Cerebellar Atrophy - Epilepsy  - Peripheral Neuropathy  - MRD9 | | Lee J.R. et al., 2015 |
|  | |  | | p.V144F  (c.430G>T) | | - Optic Nerve Atrophy - MRD9  - Peripheral Neuropathy  - Microcephaly  - Sensorineural HI | | Lee J.R. et al., 2015; Hartley et al., 2018 |
|  | |  | |  | |  | |  |
|  | | Dominant,  de novo | | p.R167C  (c.499C>T) | | - Cerebellar Atrophy  - Optic Nerve Atrophy  - Peripheral Neuropathy  - MRD9 | | Hotchkiss et al., 2016; Zhou et al., 2017 |
|  | |  | | p.G199R  (c.595G>A) | | - Cerebellar Atrophy  - Optic Nerve Atrophy  - Peripheral Neuropathy  - MRD9 | | Citterio et al., 2015 |
|  | |  | | p.A202P  (c.604G>C) | | - Optic Nerve Atrophy - Cerebral Atrophy  - Microcephaly  - MRD9 | | Lee J.R. et al., 2015 |
|  | |  | | p.S215R  (c.643A>C) | | - Cerebellar Atrophy - Optic Nerve Atrophy  - Optic Nerve Hypoplasia  - Cerebral Atrophy  - Corpus Callosum Atrophy  - Epilepsy  - Peripheral Neuropathy  - MRD9  - Microcephaly | | Lee J.R. et al., 2015; Raffa et al., 2017 |
| **Complicated SPG-30, cont.** | |  | | p.R216C  (c.646C>T) | | - Cortical Visual Impairment - MRD9 - Ataxia | | Esmaeeli-Nieh et al., 2015 |
|  | |  | | p.R216H  (c.647G>A) | | - Cerebellar Atrophy  - Corpus Callosum Hypoplasia  - Optic Nervße Atrophy  - Cerebral Atrophy  - Epilepsy  - Peripheral Neuropathy  - MRD9 | | Esmaeeli-Nieh et al., 2015; Travaglini et al., 2018 |
|  | |  | |  | |  | |  |
|  | | Dominant,  de novo | | p.R216P  (c.647G>C) | | - Cerebellar Atrophy - Optic Nerve Atrophy  - Epilepsy  - MRD9 | | Lee J.R. et al., 2015 |
|  | |  | | p.E253K  (c.757G>A) | | - Cerebellar Atrophy - Cortical Visual Impairment  - Optic Nerve Atrophy  - Peripheral Neuropathy  - MRD9  - Microcephaly | | Esmaeeli-Nieh et al., 2015 |
|  | |  | | p.R254W  (c.760C>T) | | - Ataxia - Cerebellar Atrophy  - Optic Nerve Atrophy  - Peripheral Neuropathy  - MRD9 | | Ohba et al., 2015 |
|  | |  | | p.R254Q  (c.761G>A) | | - Ataxia - Oculomotor Apraxia - MRD9 | | Ohba et al., 2015 |
| **Complicated SPG-30, cont.** | |  | | p.T258M  (c.773C>T) | | - Optic Nerve Atrophy - Corpus Callosum Hypoplasia  - Optic Nerve Hypoplasia  - Epilepsy  - Peripheral Neuropathy  - MRD9  - Microcephaly | | Cheon et al., 2017 |
|  | | Dominant,  familial | | p.R307Q  (c.902G>A) | | - Cerebellar Atrophy - Corpus Callosum Hypoplasia  - Nystagmus  - Optic Nerve Atrophy  - Epilepsy  - Peripheral Neuropathy  - MRD9  - Microcephaly | | Ohba et al., 2015; Hotchkiss et al., 2016 |
|  | | Recessive, familial | | p.A255V  (c.765C>T) | | - Peripheral Neuropathy | | Klebe et al., 2012 |
|  | |  | | p.R350G  (c.1048C>G) | | - Ataxia - Cerebellar Atrophy  - Peripheral Neuropathy | | Klebe et al., 2012 |
|  | |  | | p.S242R  (c.726C>G)  [+BEAN and TK2 mutation 🡪 SCA31] | | -Ataxia (SCA 31) - Cerebellar Atrophy - Peripheral Neuropathy - Intellectual Disability | | Hasegawa et al., 2016 |
|  | |  | | p.L249P  (c.746T>C)  [+ HUWE1 mutation] | | - Ataxia  - Cerebellar Atrophy  - Nystagmus  - MRD9  -Autism Spectrum Disorder | | Demily et al., 2018 |
|  | | Dominant, de novo | | p.R13H  (c.38G>A)  [+ LYST, PLEKHG5 and NEB mutations] | | - Autism Spectrum Disorder - Peripheral Neuropathy | | Tomaselli et al., 2017 |
|  | |  | | p.T99M (c.296C>T) | | - Cortical Visual Impairment  - Optic Nerve Atrophy  -Cerebral Atrophy  - Epilepsy  - MRD9  - Microcephaly  -Spastic Paraplegia  - Cerebellar Atrophy | | Esmaeeli-Nieh et al., 2015 |
|  | |  | |  | |  | |  |
|  | |  | | p.E148D (c.444G>T) | | - Ataxia - Cerebellar Atrophy  - Cerebral Atrophy  - Nystagmus  - Astigmatism  - MRD9  -Hypotonia -Hyperreflexia | | Ohba et al., 2015 |
| **PEHO Syndrome, cont.** | |  | | p.E253K (c.757G>A) | | - Cerebellar Atrophy - Optic Nerve Atrophy  - Cerebral Atrophy  - Epilepsy  - Peripheral Neuropathy  - MRD9  - Microcephaly  -Hypotonia | | Lee J.R. et al., 2015;  Samanta & Gokden, 2019 |
| **Unclear Hypotonia Disorders** | | Dominant, de novo | | p.S58L  (c.173C>T) | | - Cerebellar Atrophy - Peripheral Neuropathy - MRD9  -Hyperreflexia | | Megahed et al., 2016 |
|  | |  | | p.T99M  (c.296C>T) | | -Optic Nerve Atrophy -Epilepsy -Microcephaly -MRD9 -Hyperreflexia | | Esmaeeli-Nieh et al., 2015 |

$Even within the same mutation, patients have exhibited a variety of neurological conditions. For example, one patient with mutation p.T99M may exhibit ataxia while another p.T99M patient does not.

**TABLE 1 cont.**

KIF1A Mutations Existing Outside the KIF1A Motor Domain: SPG and HSAN-IIC.

| Neurodegenerative  Condition | Nature of Mutation | Amino Acid Mutation (Nucleotide) | Functional  Domain(s) Affected | Other Associated Neurological Conditions | References |
| --- | --- | --- | --- | --- | --- |
| **Pure SPG-30** |  |  |  |  |  |
|  | Dominant, familial | p.Q632*  (c.1894C>T) | CC2  CC3  PH | - | Warrenburg et al., 2016 |
| **Complicated**  **SPG-30** |  |  |  |  |  |
|  | Dominant, familial | p.I1127T  (c.3380T>C) | - | - Ataxia | Citterio et al., 2015 |
| **Unclear, Complicated SPG-30** |  |  |  |  |  |
| (p.N405Kfs*40 and p.R970H belong to a single patient.) | Recessive, familial | p.N405K  fs*40  (c.1214dup) | CC1 FHA CC2 CC3 PH | - Cerebellar Atrophy - Peripheral Neuropathy - Intellectual Disability - Psychosis | Krenn et al., 2017 |
|  |  | p.R970H  (c.2909G>A) | - | - Cerebellar Atrophy - Peripheral Neuropathy - Intellectual Disability - Psychosis | Krenn et al., 2017 |
| **HSAN-IIC** |  |  |  |  |  |
| *No Autonomic Dysfunction for either mutation. | Recessive, familial | p.L947R  fs*4  (c.2840delT) | PH | - Neuromuscular Atrophy (Feet) | Riviere et al., 2011 |
| **HSAN-IIC, cont.** |  | p.S1758Q  fs*7  (c.5271dupC) | PH | - Neuromuscular Atrophy (Feet) | Riviere et al., 2011 |

**TABLE 2**

KIF1C Mutations & Corresponding Neurological Disorders.

| Neurodegenerative  Condition | Nature of Mutation | Amino Acid Mutation (Nucleotide) | Functional  Domain(s) Affected | Other Associated Neurological Conditions | References |
| --- | --- | --- | --- | --- | --- |
| Complicated  SPG-58 |  |  |  |  |  |
|  | Recessive,  familial | Splice  (c.183G>A) | Motor Domain | - SPAX2  - Hypodontia  -Intellectual Disability | Novarino et al., 2014 |
|  |  | p.G102A  (c.305G>C)  [Patients also have p.P176L mutation.] | Motor Domain | - Ataxia  - Cerebral Atrophy  - Cerebellar Atrophy  - Dysarthria  - Peripheral Neuropathy | Oteyza et al., 2014 |
|  |  |  |  |  |  |
|  |  | p.R155*  (c.463C>T) | Motor Domain | - Ataxia  - Cerebral Atrophy  - Dysarthria  - Nystagmus  - Borderline IQ | Yücel-Yilmaz et al., 2018 |
|  |  | p.P176L  (c.527C>T)  [Patients also have p.G102A mutation.] | Motor Domain | - Ataxia  - Cerebral Atrophy  - Cerebellar Atrophy  - Dysarthria  - Peripheral Neuropathy | Oteyza et al., 2014 |
|  |  | p.R731*  (c.2191C>T) | PTPD1 Domain | - SPAX2  - Cerebral Atrophy  - Dysarthria  -Intellectual Disability  - Nystagmus | Novarino et al., 2014; Dor et al., 2014 |
|  | Dominant,  familial | p.R301G  (c.901A>G) | Motor Domain | - Dysarthria  - Peripheral Neuropathy | Oteyza et al., 2014 |
| Other KIF1C Disorders^¥^ |  |  |  |  |  |
|  | Recessive,  familial | p.R169W  (c.505C>T) | Motor Domain | -Ataxia  -Dysarthria | Dor et al., 2014 |
|  |  | p.A828Rfs*13 (c.1214dup) | TruncationOutside PTPD1 Domain | - Ataxia  - Dysarthria  - Nystagmus | Yücel-Yilmaz et al., 2018 |
|  | Dominant,  familial | p.G102A  (c.305G>C) | Motor Domain | - Cerebellar Atrophy  - Peripheral Neuropathy | Oteyza et al., 2014 |

^¥^ Other KIF1C disorders involve mutations that do not involve spasticity as a symptom. Despite this lack of spasticity, p.R169W and p.A8282Rfs*13 were reported as corresponding to complicated HSP.
